# 1342. The Utilization of Cycle Threshold Values in Hospitalized Veterans with Persistently Positive SARS CoV-2 RT-PCR for the Removal of COVID Precautions.

**DOI:** 10.1093/ofid/ofad500.1179

**Published:** 2023-11-27

**Authors:** Debra Belanger, Lisa Bailey, Mahfuz Rahman, Monique Thorne, Florence M Ford, George Psevdos

**Affiliations:** Northport VAMC, Northport, New York; Northport VAMC, Northport, New York; Renaissance School of Medicine at Stony Brook University, Corona, New York; Northport VAMC, Northport, New York; Northport VA, Northport, New York; Northport VA Medical Center, Northport, New York

## Abstract

**Background:**

In the last 3 years the novel severe acute respiratory syndrome coronavirus 2 (SARS CoV-2), which causes coronavirus disease 2019 (COVID 19), has menaced the world causing morbidity and mortality. Infection control preventionists had stepped up and were instrumental in the prevention of hospital acquired COVID infections. Discontinuation of isolation precautions in prolonged hospitalized patients and with persistently positive reverse-transcriptase polymerase chain reaction (RT-PCR) can be quite challenging. Distinguishing prolonged viral shedding vs. detection of inactive viral remnant is not easy. Cycle threshold (Ct) values can be of help, knowing that Ct values > 35 are not related with viable virus. We reviewed our experience of using Ct for infection control decisions

**Methods:**

Retrospective chart review of hospitalized Veterans (VETS) in Northport VAMC from 1/1/2021 to 3/31/23. Viral burden rebound was defined as a case of Ct showing the following trend: from low numbers to ≥35 and then decreasing to below 35. Ct values were obtained from SARS CoV-2 RT-PCR, Xpert® Xpress, Cepheid

**Results:**

2730 VETS had COVID 19 during the pandemic. Of the 235 VETS hospitalized in the study period 20 had Ct values that remains ≤ 35 for > 14 days. All were men. 80% Caucasian. The median age was 78.5. 2 were unvaccinated. 3 had COVID in the past (5, 8, 20 months prior). See Table 1. 90% had hypertension and 30% had active malignancy. The median length of stay was 23 days (16-76). The median days duration of Ct being < 35 is 20 (16-45). The lowest Ct values ranged from 16 to 29.3. In 8 patients the lowest value was on admission, in the rest it ranged from hospital day 3 to 23. 7 patients showed evidence of viral burden rebound with 1/7 having a Ct < 35 for 45 days. 2/7 had received monoclonal antibody therapies, 1 molnupiravir, and 4 remdesivir/steroids. The median duration of COVID 19 precautions was 20, range 15 to 55. 1 VET required intubation. 2 died but did not have evidence for viral burden rebound. 3 remained asymptomatic and did not need treatment

**Table 1**

**Figure:**
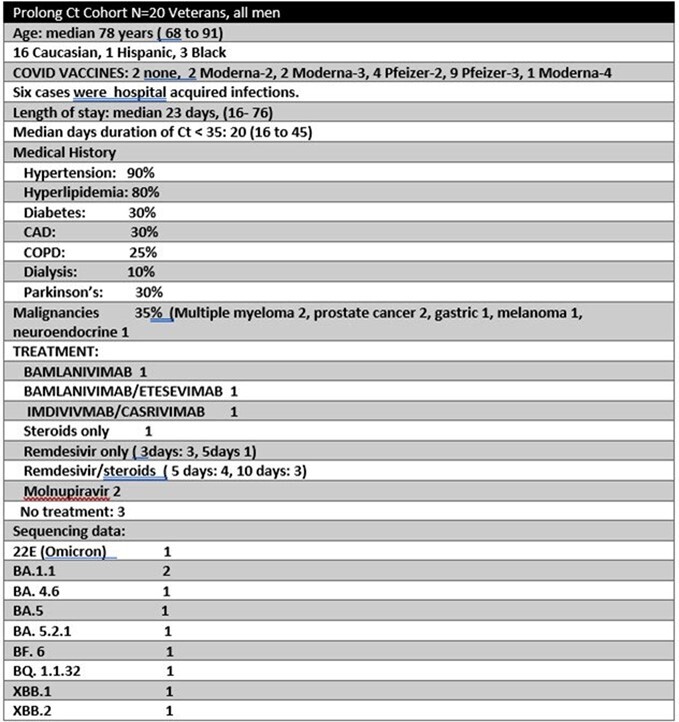

Demographics Table

**Conclusion:**

Prolongation of COVID 19 precautions for more than 14 days were noted in elderly Veterans with comorbidities, with 1/3 having active malignancy. Utilization of Ct value was crucial in identifying cases of viral burden rebound and thus extending infection control measures

**Disclosures:**

**All Authors**: No reported disclosures

